# Glucagon like peptide-1 receptor agonist therapy and long-term renal outcomes after radical nephrectomy: a propensity-matched cohort analysis

**DOI:** 10.1007/s11255-026-05177-2

**Published:** 2026-05-13

**Authors:** Noor Banihashem Ahmad, Amar Kassim, Sri Saran Manivasagam, Jay D. Raman

**Affiliations:** 1 Penn State College of Medicine, 700 HMC Crescent Road, Hershey, PA 17033 USA; 2 Department of Urology, Penn State Health Milton S. Hershey Medical Center, 500 University Drive, Hershey, PA 17033 USA

**Keywords:** Glucagon-like peptide hormone-1, Radical nephrectomy, Renal outcomes, Renal cell carcinoma

## Abstract

**Objective:**

To evaluate the association between glucagon-like peptide-1 receptor agonist (GLP-1 RA) therapy and long-term renal function outcomes following radical nephrectomy (RN).

**Methods:**

Using the TriNetX Research Network, we identified adults who underwent RN between 2005 and 2025. Patients were stratified based on GLP-1 RA use in the peri-operative period. Propensity score matching (1:1) was performed to control for demographic factors, baseline laboratory parameters, and relevant comorbidities. Baseline renal function, including serum creatinine and estimated glomerular filtration rate (eGFR), was included in the matching process. Renal outcomes included acute kidney injury (AKI), chronic kidney disease (CKD) stage 3, CKD stage 4, CKD stage 5, dialysis dependence, and eGFR, assessed at 1-, 2-, and 5-year follow-up intervals. Time-to-event analyses were conducted using Kaplan–Meier methods and Cox proportional hazards models.

**Results:**

After matching, 3316 patients (1658) per group) were included in the final analysis. GLP-1 RA users demonstrated significantly lower AKI incidence at 1 year (6.3% vs 12.4%), 2 years (9.5% vs 16.8%), and 5 years (13.4% vs 22.1%) (all p < 0.001). GLP-1 RA use was associated with significantly lower incidence of CKD Stage 3 (at 1 year (12.3% vs 17.2%), 2 years (15.% vs 21.7%, and 5 years (19.7%, vs 25.4%), CKD stage 4 at 1 year (3.1% vs 6.9%), 2 years (4.7% vs 9.0%), and 5 years (6.2% vs 10.9%), and CKD stage 5 at 2 years (1.2% vs 3.9%) and 5 years (1.7% vs 4.7%) (all p < 0.001). Dialysis dependence was also markedly reduced in the GLP-1 RA cohort (2 years: 1.0% vs 5.6%; 5 years: 1.7% vs 7.3%; all p < 0.001). Kaplan–Meier analyses demonstrated improved AKI-free, advanced CKD-free, and dialysis-free survival among GLP-1 RA users, with Cox proportional hazards models confirming significantly lower hazards of AKI, advanced CKD (stage 3–5), and dialysis dependence.

**Conclusion:**

GLP-1 RA therapy is associated with improved renal function outcomes following radical nephrectomy, with the most consistent benefit observed in reducing progression to advanced CKD (stage 3–5) and dialysis dependence. These findings support further investigation of GLP-1 RAs as a potential renal protective strategy in patients after nephrectomy.

## Introduction

Renal cell carcinoma (RCC) is a common malignancy, and radical nephrectomy is a management option for localized disease particularly for tumors not amenable to kidney preservation. However, loss of nephron mass from nephrectomy predisposes patients to chronic kidney disease (CKD), particularly in older adults or those with comorbidities [1]. Up to 48% of patients develop new or worsening CKD after radical nephrectomy, with increased risk of cardiovascular morbidity and mortality. Additionally, 43% of patients develop acute kidney injury (AKI) postoperatively [2].

Emerging pharmacologic strategies may mitigate renal decline in high-risk populations. Glucagon-like peptide-1 receptor agonists (GLP-1 RAs), initially developed for glycemic control, have demonstrated significant cardiorenal benefits in patients with type 2 diabetes [3, 4]. Large randomized controlled trials (RCTs) and meta-analyses have shown that GLP-1 RAs reduce progression to macroalbuminuria, decline in estimated glomerular filtration rate (eGFR), and need for dialysis [4–8]. Notably, a recent trial suggests these renal protective effects may extend beyond patients with diabetes and include those who are overweight or with obesity, independent of glycemic changes [4].

The biological mechanisms underlying GLP-1 RA–induced kidney protection are multifactorial and include reductions in intraglomerular pressure, inflammation, and oxidative stress, as well as natriuretic effects mediated via inhibition of the Na^+^/H^+^ exchanger in renal proximal tubules [9]. These pathways may be particularly relevant in patients post-nephrectomy, who face greater functional demands on their remaining nephrons and are more susceptible to accelerated decline [3].

Despite growing interest in GLP-1 RAs as renal protective agents, their use and effects in surgical populations remain largely underexplored. No studies to date have specifically examined GLP-1 RA use and renal function outcomes in patients who have undergone radical nephrectomy. Understanding whether these agents confer kidney benefits in this context could inform perioperative management and long-term care strategies.

In this retrospective cohort study, we examined the association between GLP-1 RA use and renal function outcomes among patients who underwent radical nephrectomy. We hypothesized that GLP-1 RA users would experience slower eGFR decline, lower incidence of AKI, and reduced progression to advanced kidney disease and dialysis dependence compared to non-users. The goal was to investigate whether GLP-1 RAs might offer a protective role in preserving renal function post-nephrectomy.

## Methods

A retrospective cohort study was conducted using the TriNetX Research Network, a user-based platform that stores de-identified electronic health records (EHRs) from participating healthcare institutions globally. The database provides access to longitudinal EHR data, including diagnoses, procedures, prescribed medications, laboratory values, and patient demographics. Only de-identified, aggregated patient-level data are made available to researchers; no protected health information (PHI) is accessible, and all analyses comply with the Health Insurance Portability and Accountability Act (HIPAA). Because the TriNetX platform only returns summary statistics using de-identified data, this study was exempt from institutional review board oversight.

The study population included individuals aged 18 and over who underwent a radical nephrectomy (RN) between 2005 and 2025. Procedures were identified using CPT codes 50,545 and 50,230, corresponding to *laparoscopic radical nephrectomy* (including removal of Gerota’s fascia and surrounding fatty tissue, regional lymph nodes, and adrenalectomy) and *open radical nephrectomy with partial ureterectomy* (including rib resection, with regional lymphadenectomy and/or vena caval thrombectomy), respectively. Patients were included in the cohort on the date of the earliest included procedure, which was treated as an index event.

Patients were stratified based on GLP-1 RA exposure during the perioperative course, defined as use at any time within the four weeks before surgery and continuing into the postoperative period. This perioperative exposure window was selected to capture active pharmacologic therapy at the time of surgery, as GLP-1 receptor agonists are typically administered weekly or daily and maintain biologic activity over several weeks. The GLP-1 RA cohort required documented use of at least one GLP-1 RA medication (liraglutide, semaglutide, exenatide, dulaglutide, albiglutide, lixisenatide, or tirzepatide), identified using RxNorm or ATC codes included in the TriNetX query. These individuals in the GLP-1 RA cohort were assumed to be regular users of the drug. Patients with prior diagnosis of chronic kidney disease were excluded to allow evaluation of incident renal dysfunction attributable to nephron mass loss following RN rather than progression of pre-existing kidney disease.

To minimize confounding, 1:1 propensity score matching (PSM) was performed via inbuilt statistical software of TriNetX. Matched variables included age, sex, race, and ethnicity, body mass index (BMI), serum creatinine, and glomerular filtration rate (GFR), and comorbidities: hypertensive disorders, ischemic heart disease, liver disease, chronic lower respiratory tract disease, cerebrovascular disease, diabetes mellitus, overweight/obesity, and tobacco use (defined by ICD-10 codes). Renoprotective medications such as angiotensin converting enzyme (ACE) inhibitors, angiotensin II receptor blockers (ARB), and sodium-glucose cotransporter II (SGLT2) inhibitors were also included in matching variables. Clinically relevant variables, including tumor size, tumor stage, surgical approach, operative time, and estimated blood loss, are not consistently captured within the TriNetX database and therefore were not included in the propensity score matching. Baseline characteristics before and after matching are demonstrated in Table 1.

**Table 1:** Baseline characteristics before and after propensity score matching

Variable	Before matching				After matching			
	No GLP-1 RA(N = 23,149)	GLP-1 RA(N = 1746)	P-value	Std diff	No GLP-1 RA(N = 1658)	GLP-1 RA(N = 1658)	P-value	Std diff
Age (years, mean ± SD)	60.4 ± 14.2	61.5 ± 10.7	0.002	0.086	61.5 ± 12.1	61.5 ± 10.8	0.918	0.004
Female	8995 (39.4%)	819 (46.9%)	< 0.001	0.152	815 (49.2%)	776 (46.8%)	0.175	0.047
Male	13,837 (60.6%)	927 (53.1%)	< 0.001	0.152	843 (50.8%)	882 (53.2%)	0.175	0.047
Black or African American	2629 (11.5%)	241 (13.8%)	0.004	0.069	234 (14.1%)	232 (14.0%)	0.920	0.003
White	16,967 (74.3%)	1284 (73.5%)	0.482	0.017	1241 (74.8%)	1217 (73.4%)	0.341	0.033
Asian	557 (2.4%)	47 (2.7%)	0.511	0.016	41 (2.5%)	47 (2.8%)	0.517	0.023
American Indian/Alaska Native	94 (0.4%)	10 (0.6%)	0.318	0.023	10 (0.6%)	10 (0.6%)	1.000	< 0.001
Other Race	691 (3.0%)	64 (3.7%)	0.136	0.036	54 (3.3%)	59 (3.6%)	0.632	0.017
Unknown Race	1814 (7.9%)	99 (5.7%)	0.001	0.090	78 (4.7%)	92 (5.5%)	0.270	0.038
Hispanic or Latino	2117 (9.3%)	160 (9.2%)	0.882	0.004	166 (10.0%)	153 (9.2%)	0.444	0.027
Not Hispanic or Latino	17,735 (77.7%)	1380 (79.0%)	0.184	0.033	1320 (79.6%)	1308 (78.9%)	0.607	0.018
AKI & CKD (N17–N19)	6135 (26.9%)	1162 (66.6%)	< 0.001	0.867	1082 (65.3%)	1082 (65.3%)	1.000	< 0.001
Hypertension (I10–I1A)	12,736 (55.8%)	1561 (89.4%)	< 0.001	0.814	1508 (91.0%)	1473 (88.8%)	0.044	0.070
Ischemic heart disease (I20–I25)	4416 (19.3%)	632 (36.2%)	< 0.001	0.383	598 (36.1%)	594 (35.8%)	0.885	0.005
Diabetes mellitus (E08–E13)	4961 (21.7%)	1355 (77.6%)	< 0.001	1.348	1270 (76.6%)	1268 (76.5%)	0.935	0.003
Obesity (E65–E68)	4868 (21.3%)	1310 (75.0%)	< 0.001	1.275	1266 (76.4%)	1222 (73.7%)	0.078	0.061
Chronic lung disease (J40–J4A)	3658 (16.0%)	568 (32.5%)	< 0.001	0.392	522 (31.5%)	526 (31.7%)	0.881	0.005
ARB use	3466 (15.2%)	673 (38.5%)	< 0.001	0.547	613 (37.0%)	620 (37.4%)	0.801	0.009
ACE inhibitor use	5106 (22.4%)	830 (47.5%)	< 0.001	0.547	765 (46.1%)	771 (46.5%)	0.834	0.007
SGLT2 inhibitor use	468 (2.0%)	396 (22.7%)	< 0.001	0.660	254 (15.3%)	314 (18.9%)	0.006	0.096
Creatinine (mean ± SD)	1.7 ± 5.7	1.8 ± 4.5	0.748	0.009	2.1 ± 2.6	1.8 ± 4.6	0.018	0.083
eGFR (mean ± SD)	68.5 ± 29.0	50.4 ± 21.0	< 0.001	0.716	56.9 ± 31.2	50.5 ± 21.0	< 0.001	0.238

Renal and metabolic outcomes were assessed at 1-, 2-, and 5-year follow-up using Kaplan–Meier analytics modules. Primary outcomes included acute kidney injury (ICD-10 N17), CKD stage 3, CKD stage 4, CKD stage 5 (ICD-10 N18.3, 18.4, and 18.5 respectively), new dialysis dependence (ICD-10 codes Z99.2, Z49 and CPT codes 90,937, 90,935, 1,012,740), creatinine, and estimated glomerular filtration rate (eGFR). For each outcome, TriNetX generated risk ratios, risk differences, odds ratios, 95% confidence intervals, and time-to-event endpoints, which were additionally evaluated using Kaplan–Meier survival curves, log-rank testing, and Cox proportional hazards models. Relative risk was calculated using GLP-1 RA users as the experimental group and non–GLP-1 RA users as the reference group.

## Results

### Baseline characteristics

Across the 1-, 2-, and 5-year analytic windows, the unmatched radical nephrectomy cohorts included approximately 23,149 patients without GLP-1 RA exposure and 1,746, patients with GLP-1 RA exposure. Prior to propensity score matching, baseline demographic differences were modest Patients in the GLP-1 RA cohort were similar in age (mean 61.5 vs 60.4 years, p = 0.002) but were more frequently female (46.9% vs 39.4%, p < 0.001). A slightly higher proportion of patients in the GLP-1 RA cohort identified as Black or African American (13.8% vs 11.5%, p = 0.004), while distributions of White race, Asian race, and Hispanic ethnicity were largely comparable between groups.

As expected, baseline comorbidity profiles showed that patients receiving GLP-1 RAs had substantially greater cardiometabolic burden. Compared with the non–GLP-1 RA group, the GLP-1 RA cohort had markedly higher prevalence of diabetes mellitus (77.6% vs 21.7%, p < 0.001), overweight/obesity (75.0% vs 21.3%, p < 0.001), and hypertensive disease (89.4% vs 55.8%, p < 0.001). They also had higher rates of ischemic heart disease (36.2% vs 19.3%, p < 0.001), chronic lower respiratory disease (32.5% vs 16.0%, p < 0.001).

Baseline use of medications known to influence renal outcomes was assessed and incorporated into the propensity score matching model. Prior to matching, patients in the GLP-1 RA cohort were significantly more likely to be prescribed SGLT2 inhibitors (22.7% vs 2.0%, p < 0.001), ACE inhibitors(47.5% vs 22.4%, p < 0.001), and ARBs (38.5% vs 15.2%, p < 0.001), reflecting differences in baseline medical management.

After propensity score matching, 3,316 patients (1,658 per group) were included in the final analytic sample. Across the 1-, 2-, and 5-year matched cohorts, age, sex, race/ethnicity, major comorbidities (hypertension, diabetes, obesity, ischemic heart disease, chronic lung disease), and available laboratory parameters were well balanced. A small residual imbalance persisted in baseline eGFR and SGLT2 inhibitors with standardized mean differences exceeding 0.10. These matched cohorts were used for all subsequent time-to-event and risk analyses. The characteristics of patients before and after matching are summarized in Table 1. After matching, no statistically significant differences between the groups persisted.

### Survival analysis and renal outcomes

Across the 1-, 2-, and 5-year follow-up windows, GLP-1 RA use was consistently associated with mitigation of loss of renal function. After propensity score matching, 3316 patients (1593 per group) contributed to the time-to-event analyses.

### Acute kidney injury (AKI)

**Fig. 1:**
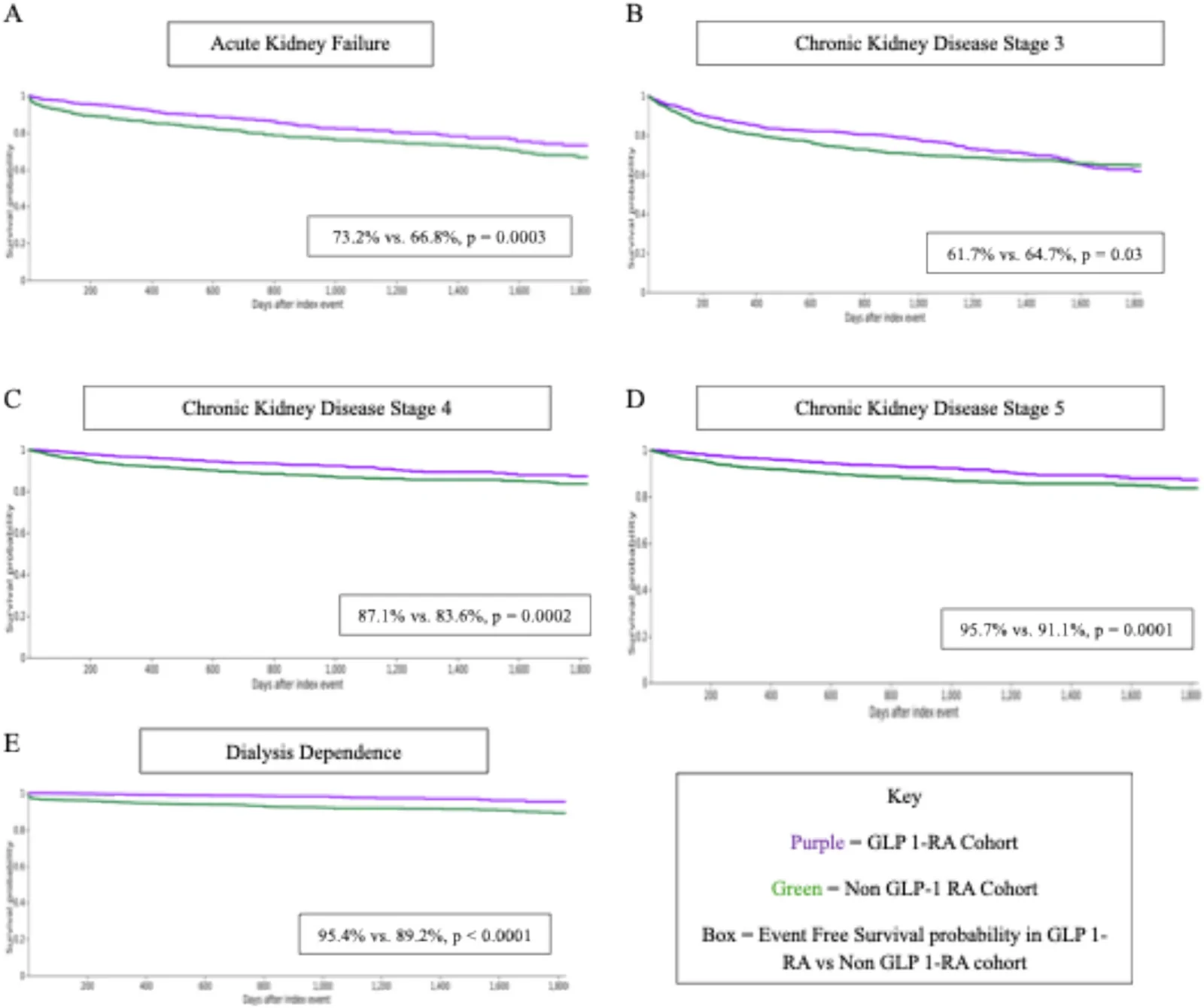
Kaplan–Meier curves demonstrating 5-year event-free survival following radical nephrectomy by GLP-1 receptor agonist (GLP-1 RA) exposure. **A**–**E** show (**A**) acute kidney injury (*AKI*), (**B**) CKD stage 3, (**C**) CKD stage 4, (**D**) CKD stage 5, and (**E**) dialysis dependence

GLP-1 RA exposure was associated with substantially lower AKI incidence at all time points. Within 1 year, AKI occurred in 6.3% of GLP-1 RA cohort vs 12.4% in non-GLP-1 RA cohort of patients (RR 0.51); within 2 years, 9.5% vs 16.8% (RR 0.57); within 5 years, 13.4% vs 22.1% (RR 0.61), all p < 0.001. Kaplan–Meier curves demonstrated improved AKI-free survival for GLP-1 RA users at each interval (1 year: 92.6% vs 86.6%; 2 years: 87.3% vs 79.8%; 5 years: 73.2% vs 66.8%, all log-rank p < 0.003), and Cox proportional hazards modeling confirmed a significantly reduced hazard of AKI associated with GLP-1 RA therapy (HR 0.67, 95% CI 0.53–0.83) at 5 years. The Kaplan–Meier Curve is shown in Fig. 1A and values are summarized in Table 2.

**Table 2:** Incidence of renal outcomes after radical nephrectomy with and without GLP-1 receptor agonist therapy

Outcome	Timepoint	GLP-1 RA users	No GLP-1 RA users	Relative risk (95% CI)	Cox hazard ratio (95% CI)
Acute kidney injury (AKI)	1 year	6.3%	12.4%	0.51 (0.38–0.68)	0.50 (0.37–0.69)
	2 years	9.5%	16.8%	0.57 (0.44–0.73)	0.56 (0.44–0.73)
	5 years	13.4%	22.1%	0.61 (0.50–0.75)	0.67 (0.53–0.83)
CKD stage 3	1 year	12.3%	17.2%	0.72 (0.53–0.86)	0.71 (0.57–0.89)
	2 years	15.0%	21.7%	0.70 (0.58–0.83)	0.69 (0.56–0.85)
	5 years	19.7%	25.4%	0.78 (0.66–0.91)	0.82 (0.66–0.98)
CKD stage 4	1 year	3.1%	6.9%	0.44 (0.31–0.63)	0.44 (0.31–0.63)
	2 years	4.7%	9.0%	0.53 (0.40–0.70)	0.53 (0.39–0.71)
	5 years	6.2%	10.9%	0.58 (0.45–0.74)	0.61 (0.47–0.80)
CKD stage 5	1 year	Not reported	Not reported	–	–
	2 years	1.2%	3.9%	0.38 (0.22–0.66)	0.39 (0.22–0.66)
	5 years	1.7%	4.7%	0.36 (0.24–0.57)	0.42 (0.27–0.66)
Dialysis dependence	1 year	Not reported	Not reported	–	–
	2 years	1.0%	5.6%	0.17 (0.09–0.30)	0.17 (0.09–0.30)
	5 years	1.7%	7.3%	0.23 (0.14–0.35)	0.26 (0.16–0.40)

### Chronic kidney disease (CKD) by stage

#### CKD stage 3

There was a statistically significant difference in CKD stage 3 incidence between groups. At 1 year, CKD stage 3 occurred in 12.3% of GLP-1 RA users compared with 17.2% of non-users (RR 0.72). At 2 years, incidence was 15.0% vs 21.7% (RR 0.70), and at 5 years, 19.7% vs 25.4% (RR 0.78). Hazard ratios were statistically significant across timepoints (0.71, 0.69, 0.82 respectively). Results are summarized in Table 2. Kaplan–Meier estimated survival at the end of follow-up was 61.7% in the GLP-1 RA group and 64.7% in the non–GLP-1 RA group. Despite this, time-to-event analysis demonstrated a significantly lower hazard among GLP-1 RA users (HR 0.82, p = 0.0001), with a corresponding difference between survival curves (log-rank p = 0.032).

#### CKD stage 4

GLP-1 RA use was associated with significantly lower incidence of CKD stage 4 at all timepoints. At 1 year, CKD stage 4 occurred in 3.1% of GLP-1 RA users compared with 6.9% of non-users (RR 0.44). At 2 years, incidence was 4.7% vs 9.0% (RR 0.53), and at 5 years, 6.2% vs 10.9% (RR 0.58), all p < 0.001. Cox proportional hazards modeling demonstrated a reduced hazard of CKD stage 4 among GLP-1 RA users (HR 0.44 at 1 year; HR 0.53 at 2 years; HR 0.61 at 5 years).

#### CKD stage 5

CKD stage 5 outcomes were not reported at 1 year due to insufficient data within the TrinetX platform. At 2 years, CKD stage 5 occurred in 1.2% of GLP-1 RA users compared with 3.9% of non-users (RR 0.38), and at 5 years, 1.7% vs 4.7% (RR 0.36), both p < 0.001. GLP-1 RA use was associated with significantly reduced hazard of CKD stage 5 (HR 0.39 at 2 years; HR 0.42 at 5 years). The Kaplan–Meier curves for CKD stages 3–5 are shown in Fig. 1B–D, and corresponding outcome are summarized in Table 2 at 5 years.

### Dialysis dependence

GLP-1 RA therapy demonstrated for a strong association with decreased long-term dialysis dependency. Dialysis dependence at 1 year was not reported due to insufficient data within the TriNetX platform. At 2 years, dialysis initiation occurred in 1.0% vs 5.6% of patients (RR 0.17), and at 5 years, 1.7% vs 7.3% (RR 0.23), both p < 0.001. GLP-1 RA users compared with non-GLP-1 RA patients (2-year survival: 98.7% vs 93.6%; 5-year survival: 95.3% vs 89.2%, log-rank p < 0.003). Cox proportional hazards modeling demonstrated a markedly reduced hazard of dialysis dependence among GLP-1 RA users (2-year HR 0.11, 95% CI 0.06–0.21; 5-year HR 0.26, 95% CI 0.16–0.40). In adjusted Cox analysis, GLP-1 RA exposure remained a strongly associated factor of reduced dialysis dependence (HR 0.40, 95% CI 0.32—0.49). The Kaplan–Meier Curves are shown in Fig. 1E and values are summarized in Table 2.

### Post operative lab values

Laboratory distributions were consistent with more advanced kidney disease in the non–GLP-1 RA cohort, with higher mean serum creatinine and lower mean eGFR, whereas patients in the GLP-1 RA cohort had lower creatinine and higher eGFR at each analytic window (41.1 vs 47.8 mL/min/1.73m^2^ at 1 year, p = 0.001; 41.8 vs 48.7 mL/min/1.73m^2^ at 2 years, p = 0.001; and 41.0 vs 48.2 mL/min/1.73m^2^ at 5 years, p < 0.001**)**. Mean creatinine was significantly higher in the non-GLP-1 RA group as compared to GLP-1 RA users across the three-time intervals analyzed (2.77 vs 1.71 mg/dL at 1 year, 2.5 vs 1.8 mg/dL at 2 years, and 2.5 vs 1.7 mg/dL at 5 years, all p < 0.001).

## Discussion

In this retrospective cohort of patients undergoing radical nephrectomy, we found that GLP-1 RA use is associated with attenuated decline in renal function, lower incidence of dialysis dependence, and reduced postoperative AKI rates. These findings align with accumulating evidence that GLP-1 RAs may confer renal protective benefits, even beyond glycemic control [4–6].

Large, randomized trials, such as LEADER, have shown that GLP-1 RAs reduce the risk of macroalbuminuria and slow eGFR decline in patients with type 2 diabetes [5]. More recently, the SELECT trial demonstrated that semaglutide reduced major renal events in overweight individuals without diabetes [4]. Meta-analyses of GLP-1 RA trials consistently report reductions in composite renal outcomes, including need for dialysis, GFR decline, or kidney-related death [7, 14]. These findings are further supported by the AWARD-7 trial, in which dulaglutide preserved kidney function more effectively than insulin glargine, with higher eGFR at 52 weeks and fewer patients progressing to end-stage renal disease [15]. Similarly, other studies have demonstrated that AKI after major surgery leads to sustained loss of kidney function and higher mortality, while another reported that GLP-1 receptor agonists can improve renal outcomes through mechanisms that directly support long-term kidney function [16, 17].

Our findings extend these observations to a surgical cohort of patients with resultant solitary kidney following nephrectomy; a population that has been underrepresented in prior studies. Recent observational data further support the use of GLP-1 RAs in preventing CKD across diverse patient populations. Notably, benefits have been observed even in patients of advanced age or with pre-existing renal impairment [10, 11, 18].

Mechanistically, GLP-1 RAs may protect renal function through multiple pathways. These agents promote natriuresis, lower glomerular hypertension, and reduce inflammation and oxidative stress, partly through weight loss and metabolic effects [9, 13, 19]. GLP-1 receptors are expressed in renal proximal tubules, and GLP-1 RA therapy has been shown to inhibit sodium-hydrogen exchange, leading to improved intraglomerular hemodynamics [9, 19]. These effects may explain the slower eGFR decline observed in GLP-1 RA users in our study.

Our analysis suggest that GLP-1 Ras are associated with reduced risk of renal function decline across multiple stages of kidney disease. The benefit is most pronounced in advanced kidney disease and dialysis dependence compared to earlier-stage disease.

Although our propensity-matched cohorts balanced major cardiometabolic comorbidities, many GLP-1 RA users had underlying diabetes and obesity prior to matching, suggesting that patients with higher metabolic burden may derive greater benefit. This is biologically plausible, as GLP-1 receptor agonists improve glycemic control, promote weight loss, and reduce inflammation and blood pressure, all of which contribute to renal protection [5, 8]. Patients with multiple metabolic comorbidities may therefore be at higher baseline risk for renal decline after nephrectomy and may benefit more from these effects. However, due to limitations of the TriNetX platform, this study did not perform subgroup analyses to confirm this, and these observations should therefore be interpreted as hypothesis-generating. Future studies are needed to better identify which patient populations benefit most from GLP-1 RA therapy in the perioperative setting.

Prior observational studies have similarly reported that GLP-1 RA use is associated with lower rates of major adverse kidney events and end-stage kidney disease (ESKD) [10, 11]. Additionally, GLP-1 RAs have demonstrated a favorable safety profile in clinical trials, including a low incidence of anti-drug antibody formation [12].

After propensity matching, baseline comorbidities and pharmacotherapies that may affect outcomes were overall well balanced between GLP-1 RA users and non-users. In this context, the association between GLP-1 RA use and mitigated loss of renal function suggests a possible therapeutic benefit independent of baseline CKD risk. The reduction in postoperative AKI is notable given its known association with long-term CKD after nephrectomy [12, 16].

Emerging data are in favor of the protective effects of GLPs in numerous areas, such as glucose lowering, oxidative stress and inflammation, endothelial function, blood pressure, dyslipidemia, and weight management [17]. While GLP-1 RAs are not yet standard in perioperative protocols, their role in preserving renal function post-surgery and potentially influencing oncologic outcomes warrants further exploration [20, 21].

Limitations of our study include its retrospective design and potential for residual confounding. The use of the TriNetX database limited the availability and granularity of certain clinical variables, including medication adherence, dosage, duration of GLP-1 receptor agonist therapy, and albuminuria measurements. Pertinent metabolic parameters such as burden of metabolic co-morbities and glycemic control are not adequately captured in the database which precludes subgroup analysis to determine whether certain patient groups (i.e. those with obesity or hyperglycemia) derive additional benefit from GLP-1 RA therapies. Additionally, key surgical and oncologic variables, including tumor size, tumor stage, operative time, estimated blood loss, and histopathologic subtype, were not available within the dataset and therefore could not be accounted for in the analysis. Our study was specifically designed to evaluate outcomes following radical nephrectomy as a surgical cohort; therefore, surgical approach was beyond the scope of this analysis. Lastly, the platform does not allow for evaluation of a potential dose–response relationship in regard to GLP-1 RA medications, and our analysis was limited to class-level effects; comparison between individual medications was beyond the scope of this study and would have reduced cohort sizes, limiting the statistical robustness.

Outcomes were identified using ICD diagnostic codes, which may lack clinical granularity. In addition, AKI events were captured through diagnostic coding across follow-up intervals rather than precise postoperative timing, which may limit interpretation of early perioperative kidney injury. Additionally, we adjusted for the use of renoprotective medications, including SGLT2 inhibitors, ACE inhibitors, and ARBs, medication exposure in the TriNetX is based on coding and prescription data and does not account for adherence, duration, or timing of therapy. As a result, residual confounding from concomitant medication use cannot be fully excluded. The absence of chart-level data also limited our ability to control for confounders such as medication dose and duration. Nonetheless, our findings remain consistent with prior clinical and mechanistic studies suggesting potential renoprotective effects of GLP-1 RA following nephrectomy. Assessment of CKD stage transitions would further clarify the clinical significance of these findings but could not be evaluated within the TriNetX platform. Prior nephrectomy studies suggest that early postoperative renal decline becomes particularly consequential when patients progress to more advanced CKD stages [22], supporting the clinical relevance of the lower AKI rates and more favorable postoperative creatinine and eGFR values observed in our GLP-1 RA cohort [23]. Given these limitations, our findings should be interpreted as associative rather than causal. Prospective studies with granular clinical, pharmacologic, and surgical data are needed to validate these findings and to better define the role of GLP-1 receptor agonists as a renoprotective strategy following nephrectomy.

## Conclusion

In a large multi-center cohort, we observed that GLP-1 RA therapy after radical nephrectomy was associated with reduced long-term risk of AKI and significantly lower progression to advanced kidney disease, including CKD stage 3, 4, and 5, and dialysis dependence.. These findings suggest that GLP-1 RAs may attenuate renal decline following nephrectomy. Given the high incidence of CKD following nephrectomy, our results support prospective investigation of GLP-1 RAs as a renal protective strategy in this population. GLP-1 agonists could potentially represent a novel therapeutic approach to preserve renal function in patients after nephrectomy.

## Data Availability

The data is only available to participating institutions of TriNetX.
